# Pharmacy

**Published:** 1837-10

**Authors:** 


### PHARMACY.

*New Preparation of Opium.* By JOSEPH HOULTON, Esq.

"THE preparation, which I label *Liquor Opii*, is made as follows:—Take two ounces and a half of the best Turkey opium; thirty-two fluid ounces of Beaufoy's acid, the strength of pickling vinegar: macerate with a gentle heat for six days, frequently shaking the vessel; then filter, and evaporate the fluid to the consistence of the extracts of the Pharmacopœia, finishing the evaporation by the spontaneous method. (This I employ under the name of *Extractum Opii Aceticum.)*

"Take the above extract; alcohol, five fluid ounces; distilled water, thirty-five fluid ounces. Macerate for eight days, and filter.

"This liquor opii will be about the strength of tinctura opii in sedative property; but the strength may be varied at the will of the apothecary. I prefer it of the proportions I have stated.

"From my own observations, which have been extensive, I feel assured that it is in no respect inferior to the Liquor opii sedativus of Battley." *Med. Gaz. Aug.* 12.

